# Tug of War Over Power: A Case Study on the Development of Professional–Informal Caregiver Tensions in Residential Dementia Care

**DOI:** 10.1111/nin.70068

**Published:** 2025-11-18

**Authors:** Marleen D. W. Dohmen, Charlotte van den Eijnde, Tineke A. Abma, Barbara C. Groot, Johanna M. Huijg

**Affiliations:** ^1^ Leyden Academy on Vitality and Ageing Leiden the Netherlands; ^2^ Public Health and Primary Care Leiden University Medical Centre Leiden the Netherlands; ^3^ Socio‐Medical Sciences Erasmus School of Health Policy & Management Rotterdam the Netherlands; ^4^ Vrije Universiteit Amsterdam Amsterdam the Netherlands; ^5^ Amsterdam University of Applied Sciences Amsterdam the Netherlands

**Keywords:** care ethics, family caregivers, professional–informal caregiver relationship, relational care, residential dementia care, single case study

## Abstract

Effective collaboration between professional and informal caregivers is essential in residential dementia care, but often disrupted by relational tensions. This 2‐year case study explores how these tensions emerge and develop. Using visual and inductive analysis of qualitative data, we study a strained professional–informal caregiver relationship in a Dutch residential dementia care facility, focusing on the personal, relational, and organizational factors that shape it. Findings indicate that divergent conceptualizations of good care, rooted in distinct ethical frameworks, lead to relational tensions between professional and informal caregivers. These tensions tend to escalate through a series of interrelated dynamics that ultimately result in a tug of war over power, which triggers a downward spiral in the relationship. Organizational processes such as pseudo‐participation and unintentional support for distancing practices further entrench these dynamics. Professional and informal caregivers are prompted to work around rather than resolve the conflict. Set within a Dutch residential dementia care context, the study offers broader implications for similar care settings globally. It advocates a shift from person‐centered care to relational care, emphasizing multidirectionality to address power asymmetries. Key implications include promoting relational care as the moral standard, implementing organizational changes to support relational care and embedding moral skills into nursing education.

## 1 Introduction

Moving into a residential care facility is often a deeply emotional experience, both for new residents and their significant others. During the transition, residents may face a loss of agency, social connectedness, and sense of home (O'Neill and Ryan 2023), while their significant others may experience a complex mix of guilt, grief, and relief (Afram et al. 2015; Robinson and Fisher 2023).

Alongside these emotional adjustments, the move to a residential care facility also means reshaping caregiving roles and responsibilities. Traditionally, it involved a near‐complete handover of care from significant others to care professionals. Today, this is no longer the norm. Current practice increasingly emphasizes the active involvement of significant others as informal caregivers. First, because their personal relationship to the resident can help enrich person‐centered care (i.e., care that resonates with the resident's unique needs and wishes; Kitson et al. 2013; McCormack et al. 2011). Second, their active involvement is needed to support the sustainability of long‐term care amidst workforce shortages (Dutch Ministry of Public Health, Welfare, and Sports 2022; Dutch Care Institute 2024) affecting many Western countries.

As informal caregivers assume more active roles in care, their collaboration with professional caregivers becomes an integral part of the care process. Consequently, fostering this relationship is crucial to ensure high‐quality care for residents. This may be particularly important in residential dementia care settings. While person‐centered care is a guiding principle in these settings, people with dementia often have difficulty expressing their needs and wishes. This leads both professional and informal caregivers to act as proxy‐decision makers, interpreting what constitutes good care on the resident's behalf. However, their perspectives on good care tend to differ in practice, leading to relational tensions between them.

Another disconnect exists around the appropriate division of roles and responsibilities in the care process, about which professional and informal caregivers often have differing ideas. For example, guided by values such as expertise and responsibility, care professionals tend to assert decision‐making authority, while informal caregivers report feeling unrecognized as equal partners in care (Brouwer et al. 2005; Dohmen et al. 2025). Moreover, these perspectives are rarely made explicit, leading to ambiguity about the nature of the collaboration, which forms a key source of tension in the relationship between professional and informal caregivers (Baumbusch and Phinney 2014; Dohmen et al. 2025; Hengelaar et al. 2018; Wittenberg et al. 2018).

The relational dynamics that arise from these disconnects may unintentionally sideline the resident, leading to their passive rather than active involvement in the care process, ultimately undermining person‐centered care (Dohmen et al. 2025; Novy et al. 2023). As such, a good collaborative relationship between professional and informal caregivers is not merely desirable, but necessary to achieve mutual alignment around the shared goal of supporting the resident's well‐being through nursing practice.

While the value of effective collaboration between professional and informal caregivers has been widely acknowledged in practice, policy, and research, our understanding of how these relationships develop over time, particularly within the context of residential dementia care, remains limited. Given that a collaborative relationship is an inherently dynamic process, susceptible to change over time, the missing temporal dimension represents a notable gap in nursing literature. This gap limits our ability to support and strengthen collaboration between professional and informal caregivers to offer high‐quality care to residents with dementia.

To add to the knowledge on this topic, we conducted a single case study of a strained relationship between a team of care professionals and an informal caregiver in a Dutch residential dementia care facility. We explored their relational dynamics, and the personal, relational, and organizational factors that shape it. We draw on 2 years of qualitative descriptive data from journal entries, interviews, observations, focus groups, and digital communication, and use visual and inductive analysis and data triangulation to integrate multiple perspectives into a coherent narrative. Our findings offer deeper insight into the relational dynamics at play and may inform nursing strategies that promote stronger collaboration between professional and informal caregivers, ultimately supporting high‐quality dementia care.

## 2 Methods

### 2.1 Design

To examine how relational strains between professional and informal caregivers develop, influenced by personal, relational, and organizational factors, we adopted a single case study design. This type of design is especially suitable when phenomenon and context cannot be clearly distinguished (Green and Thorogood 2018). Inspired by naturalistic case study approaches, we conceptualize our case as embedded in a larger context (Abma and Stake 2014; Simons 2009), aligning with our aim to provide an integral understanding of professional–informal caregiver collaboration.

### 2.2 Research Team and Philosophical Underpinnings

The study was conducted by an interdisciplinary research team with expertise in nursing, care ethics, psychology, sociology, occupational therapy, participatory research and communication science. In addition, one of us is also an informal caregiver for her parents, one of whom lives in a long‐term care facility. As a team, we worked from a social constructivist and care ethics perspective. Social constructivism emphasizes the idea that knowledge is not objective or preexisting but is constructed through interactions, experiences, and contexts (Gergen and Gergen 1988; Lincoln and Guba 1985). Care ethics is a relational and contextual approach to ethics that is grounded in care practice. Rather than relying on universal moral principles, care ethics argues that notions of moral goodness (e.g., good care, good collaboration) emerge through social processes within the relationships between those involved, paying particular attention to power dynamics and human interdependence (Tronto 1993, 2013; Walker 2007).

### 2.3 Case Introduction

Over the past 4 years, we have conducted several research projects on the collaboration between professional and informal caregivers in residential care facilities. In one of those projects, we identified the case we present here. The case centers on the strained relationship between a team of care professionals at a small‐scale living facility for people with dementia in the Netherlands and a woman named Cara (pseudonym), who is involved as an informal caregiver for her mother Alma (pseudonym), a resident at the facility.

Alma lives in a unit that accommodates eight residents. The care professionals work together in a team of seven to provide daily care for these residents, with two to three professionals present per shift. The team is self‐guiding, supported by a location manager. One of the care professionals, Debbie (pseudonym), is Alma's primary caregiver and is responsible for coordinating her care and maintaining communication with Cara. The facility is part of a larger care organization that emphasizes tailoring care to individual needs through collaboration between the care recipient, their informal network, and the organization's care professionals. This vision aligns with Dutch national care policy, which promotes person‐centered care and the active involvement of informal caregivers.

### 2.4 Case Selection

The case was selected for its typicality, reflecting common real‐world situations, challenges and dynamics in the collaboration between professional and informal caregivers in Dutch residential care (i.e., typical case). Additionally, the case was chosen for its learning potential (Abma and Stake 2014), as its intensity in terms of tension and conflict illuminated relational dynamics. Moreover, the conflict presented in the case occurred during the study period. This cannot be orchestrated, creating a unique opportunity to examine the evolution of relational strains over time.

### 2.5 Case Database

Data were collected as part of a broader research project, within which the current case was identified. The complete dataset from this project was reviewed to assess its relevance to the present case study. Data were deemed relevant if they provided information about the relationship between the care professionals and Cara. Relevant data were compiled in a case database, consisting of interview transcripts, journal entries, field notes, focus group transcripts, and digital communication trails. Table 1 provides an overview of the included data and its distribution over time, specified per participant.

**Table 1:** Overview of data included in the database and its distribution over time, specified per participant.

Data source	Participant^a^	Care professionals							Informal caregiver	Location manager
		Debbie (Alma's primary caregiver)	Adriana	Caroline	Marge	Linda	Sabrina	Daisy	Cara (daughter of resident Alma)	Leandra
Journal entries^b^		Unknown	Unknown	Unknown	Unknown	Unknown	Unknown	Unknown	x	
Interviews Feb–March 2021		x	x							
Interviews April–May 2021		x	x	x	x				x	
Interviews January 2022		x	x	x	x	x	x			
Focus groups June 2021		x	x	x	x	x			x	
Digital communication		x							x	x

^a^ All names are pseudonymized.

^b^ The broader research project's design required anonymity for the journal entries of the care team. Therefore, it was not possible to specify which care professional(s) contributed to the journal entries included in the database.

#### 2.5.1 Journal Entries

Participants wrote digital journal entries about their personal experience with work, care, and life at the facility. The care professionals shared these entries anonymously, making 349 entries in total. Of these entries, eight related to their contact with Cara and were thus included in the database. Cara wrote 23 entries, all of which were included in the database.

#### 2.5.2 Qualitative Interviews

Interviews were conducted at three time points. First, we conducted individual interviews with care professionals in February and March 2021 as an interim evaluation of the broader research project. Topics included reflections on writing journal entries, its impact on their work, their professional goals, and factors that helped or hindered achieving these goals. Second, we conducted individual interviews with care professionals and informal caregivers in April and May 2021, focusing on their views of good care and collaboration, and their current experiences of working together. Finally, we conducted duo‐interviews with care professionals in January 2022 as the broader project's final evaluation. These covered similar topics as the interim interviews, with the focus on final reflections. All interviews lasted 45–60 min and were recorded and transcribed verbatim. Of the 30 interviews conducted, 10 were deemed relevant to the case and thus included in the database. These comprised nine interviews with care professionals (*n* = 6) and one with Cara.

#### 2.5.3 Field Notes

Throughout the course of the study, the researchers kept field notes on their observations. Field notes on the relationship between the care professionals and Cara were included in the database.

#### 2.5.4 Focus Groups

In June 2021, we conducted two focus groups: one with care professionals (*n* = 5) and one with informal caregivers (*n* = 3, including Cara). The discussions explored the potential value of exchanging journal entries to support collaboration (a key aim of the broader research project). Topics included participants' experiences with the exchange, its impact on their collaborative relationships, and how a meaningful process might be organized. The focus groups lasted 60–90 min and were recorded and transcribed verbatim. We planned to conduct another focus group, involving both professional and informal caregivers. However, this did not materialize as the care professionals were unwilling to participate, citing relational tensions with Cara.

#### 2.5.5 Digital Communication Trails

Included in the database were e‐mails between Cara, a care professional, the location manager, and researcher M.D.W.D., as well as organizational communications about the facility's mission, vision, and practices.

### 2.6 Data Analysis

**Figure 1:**
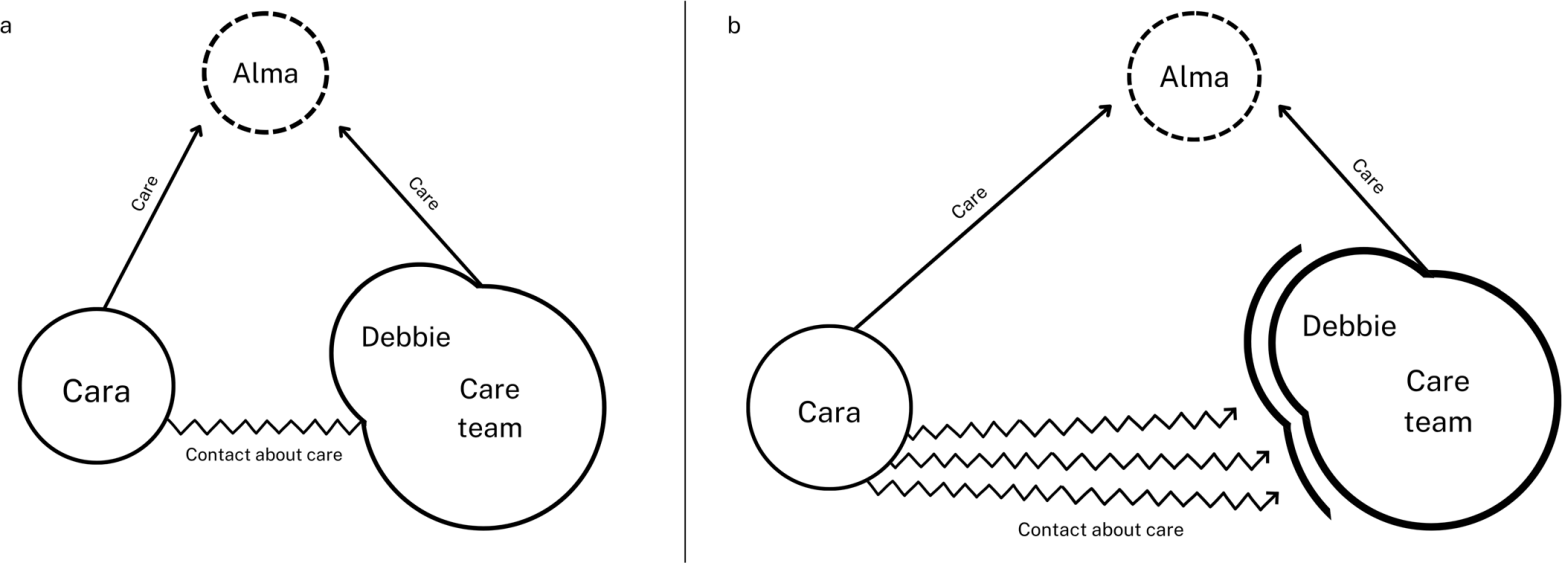
Example of the visual analysis: the development of the triadic relationship over time (a, b).

In line with our social constructivist paradigm, knowledge was construed through an inductive analysis of data within its context, integrating multiple perspectives into a coherent narrative via data triangulation. First, we familiarized ourselves with the data (Green and Thorogood 2018) by organizing the information chronologically and categorizing it into different arrays. To deepen our understanding, we also created visual representations of the relational dynamics. An example of this visual analysis is presented in Figure 1. Together, these steps helped us move towards an analytical strategy in a data‐driven way and formed the start of our iterative process, in which we combined data interpretation with systematic coding (Abma and Stake 2014).

Researchers M.D.W.D. and C.v.d.E. individually performed open coding on the data using MaxQDA version 24.7 (VERBI Software, 2024). Subsequently, they discussed and compared findings, arranging codes into themes and subthemes (i.e., axial coding). They then linked themes and subthemes to the timeline of the case, gaining insight into the development of the relationship over time. Throughout this process, findings were shared and discussed with researchers B.C.G., T.A.A., and J.M.H., who acted as “critical friends,” challenging interpretations and supporting reflexive thinking (Costa and Kallick 1993; Nishida et al. 2025).

### 2.7 Ethics and Quality

The study was reviewed and approved by the Institutional Review Board of the Medical Ethical Committee Leiden‐Den Haag‐Delft for observational studies.

The broader project in which we identified the case took place during the COVID‐19 pandemic, which involved varying mitigation measures such as visiting restrictions. These restrictions affected both the case itself and our research process. Specifically, these mitigation measures limited direct contact with the residents to prevent potential infection. As a result, we were not able to gain insight into Alma's perspective on the relational dynamics.

Given the ethical and privacy‐related sensitivity of the case, the research team engaged in continuous reflection on how to responsibly conduct and present the study. To support reflexivity, researchers M.D.W.D. and C.v.d.E. kept reflective diaries, and the team regularly reviewed assumptions, assessing how they could impact the various phases of the research process (Råheim et al. 2016). In interpreting the data, we made a conscious effort to avoid taking sides in the conflict, working from a position of multiple partiality (Abma 2006). To enhance confidentiality, names were pseudonymized and certain details were changed to protect identities, without affecting key aspects of the case.

While rooted in the Dutch context, the case's typicality offers insights for similar person‐centered care settings globally (Abma and Stake 2014; Simons 2015). Prolonged engagement with the study context and data triangulation strengthen the study's confirmability and contribute to its transferability by providing a rich context for thick description (Abma and Stake 2014; Kroopnick 2013).

## 3 Results

Below, we present our findings in two parts. In part one, we introduce the perspectives of the team of care professionals and Cara on good care and collaboration, as well as the context in which this takes place. In part two, we outline the dynamics of their relationship, affected by personal, relational, and organizational factors. Presented in chronological order and illustrated through concrete examples, the findings add to the understanding of how relational strains between the care professionals and Cara develop over time.

### 3.1 Introducing the Perspectives and the Context

#### 3.1.1 Who Knows (What Is Best for) Alma?

Following a person‐centered approach, the care professionals see good care as enhancing well‐being by tailoring care to a resident's current needs and wishes. Given their professional expertise and daily interactions with the residents, the care professionals believe that they know the residents “through and through” (Debbie, interview, May 2021) and that they are best equipped to determine how to contribute to their well‐being. Here, the changes caused by dementia play a key role. According to care professionals, significant others knew the resident as they once were, while they know the resident as they are now: “The ideal situation would be if the family stopped focusing so much on the past and focused more on how they [the residents] are now” (Debbie, interview, May 2021).

Cara believes that the care for her mother Alma should reflect her identity, that is, how Cara has known her mother throughout her life. Following Cara, this can be achieved by helping Alma preserve some of her uniquely personal traits and habits, such as having the radio tuned to a jazz station: “My mother was addicted to the radio. She used to say jazz music was her way of surviving” (journal entry, February 2021). Cara feels this is the best way to contribute to Alma's well‐being, as it ensures that she can continue living her life as she knows it. In this, she positions herself as an advocate for her mother's identity.

Both the care professionals and Cara aim to foster Alma's well‐being by advocating for her wishes and desires. In this process, Alma herself does not appear to be actively involved.

#### 3.1.2 Who Are We Here for?

The care professionals articulate their core mission as directly contributing to the well‐being of residents. While they have empathy for the emotional challenges faced by informal caregivers when a loved one is affected by dementia, care professionals believe that emotions such as worry, grief, and loss may cloud informal caregivers' judgment and ultimately hinder the resident's well‐being. They therefore expect informal caregivers to set these emotions aside. Additionally, they view this emotional restraint as the informal caregivers' own responsibility, as their primary mission is to support the resident: “I know we need family, of course, but when it comes to care, the resident is our focus. That's my job. Family is kind of a secondary thing” (Marge, focus group, June 2021).

For Cara, emotions such as worry, grief, and loss are inextricably linked to her involvement in the care for her mother. Stemming from love and connectedness, she experiences grief over the loss of what made their mother–daughter relationship special: “And calling each other at night, that tradition is also gone now. She doesn't even answer the phone anymore” (e‐mail to researcher, May 2021). Having to set these emotions aside would reduce Cara's relationship with her mother from a deeply emotional one to a more functional one, focused on fulfilling tasks to achieve well‐being for Alma. Moreover, to Cara, these emotions do not hinder her provision of good care for Alma; rather, they enable it. As an embodiment of the value of love, Cara's emotions are precisely what make her involved and engaged in caring for her mother.

#### 3.1.3 Who Is in Charge?

Organized in a self‐managing team, the care professionals are in charge of the daily routines and the care provided in their unit at the living facility. A location manager is involved, but she is mainly responsible for the organizational matters of the facility as a whole. Resultingly, the care professionals tend to experience the living unit they work on as “theirs,” asserting authority over decisions. Although there is no formal hierarchy within the team, an informal leadership structure does exist, characterized by several leaders who hold considerable influence over the team's collective mindset about good care and collaboration.

Cara is a visitor to her mother's living unit. As an advocate for her mother, she wants to have a say in her mother's care process. However, Cara describes experiencing limited space for her input ever since her mother's move to the facility. She gives an example of how Alma's intake conversation could not be rescheduled, even though this meant that it had to take place without one of Alma's significant others present. Consequently, Cara did not get the opportunity to express preferences and concerns at the beginning of the care relationship, which made her feel disregarded as a valid and valuable source of knowledge:

I really didn't have a positive or warm experience with the move to the home at all: it was decided in a very dominant way, when I had to be there for the “welcome meeting” with the residential care doctor. […] Despite my best efforts, I wasn't able to make it in time for the meeting on that day. But apparently, that was “not that important.”

(journal entry, May 2021)

#### 3.1.4 Who Has a Voice?

To promote the well‐being of residents, the care professionals place strong emphasis on achieving alignment on the best approach to care. This alignment is pursued both within their team and with informal caregivers. However, in their interactions with informal caregivers, the care professionals tend to frame alignment as agreement from informal caregivers with their professional perspective. They often aim to convince informal caregivers, rather than engage in a reciprocal exchange of ideas: “In the desired situation, staff and relatives would be on the same page. Meaning that relatives would be at peace with how we organize care” (Debbie, interview, May 2021). Within this framework, a “good” informal caregiver is implicitly defined as one who affirms and supports the care team's decisions. If informal caregivers have a different understanding of what constitutes good care, the care professionals tend to attribute this to a lack of knowledge about dementia:

The family has no idea how dementia progresses. So then you find yourselves in opposition. […] even if you explain it to them, they won't accept it. I mean, I won't say we know everything, but we are of course specialized in this disease.

(Debbie, interview March 2021)

Grounded in their professional expertise, the care professionals claim a position of authority over the care process. This authority is further reinforced by their “insider” position at the facility, which provides them with detailed, day‐to‐day knowledge of residents' well‐being. The care professionals not only possess this valuable knowledge but also control the flow of information to informal caregivers. They manage the information infrastructure used to share information with informal caregivers and curate the electronic care reports, for instance, omitting information if they expect it might upset or frustrate informal caregivers.

The care professionals generally consider information‐sharing with informal caregivers as a favor, rather than part of a reciprocal relationship. They do not particularly expect feedback or dialogue, especially not via the electronic report system. If informal caregivers do wish to provide input or feedback, the care professionals consider face‐to‐face interactions to be the only truly appropriate channel. In this way, the care professionals maintain control over communication, which tends to be one‐directional.

For Cara, there is no framework structurally organizing space for her voice. Resultingly, if Cara wants to give input or feedback, she will need to initiate contact with the care professionals. As a visitor to the facility, she experiences this as difficult. Moreover, the norm of a face‐to‐face conversation being the appropriate way to give input limits Cara's voice in the care process to the moments she visits the facility.

In an attempt to gain space for her voice, Cara has joined the location's patient council. However, she has experienced this as a fruitless endeavor and an ineffective platform for bringing about meaningful change: “I find the patient council time‐consuming and futile, actually. It seems more suited for those who like to feel important and have a fondness for rules and protocols” (journal entry, May 2021).

### 3.2 Dynamics in Interaction

#### 3.2.1 From Diverging Perspectives to a Strained Relationship

Bringing these diverging perspectives in interaction, the relationship between the care professionals and Cara turns strained. This can be illustrated by the example of Alma's dental prosthetics. Cara thinks it is important that Alma wears these prosthetics, not only for medical reasons, but also because Alma always found it important to look well‐groomed. In a journal entry, Cara writes about how her mother does not feel well without her teeth, illustrating how she keeps an eye on the continuation of her mother's identity and dignity:

And I had my mother on the phone once, and I heard her say, brought it up all on her own, that she doesn't feel well, without her dentures… It's so sad too!

(journal entry, May 2021)

However, most of the care professionals think wearing the prosthetics does not contribute to Alma's well‐being:

Alma came to have lunch in the shared kitchen. While eating, she took out her dentures and placed them on the edge of her plate. She said it hurt.

(Journal entry, shared anonymously by one of the care professionals, April 2021)

Although they understand that Alma may have found it important to look neat and tidy in the past, their day‐to‐day experience suggests this is no longer the case. This highlights their focus on tailoring care to residents' current preferences. Cara closely monitors the execution of care agreements regarding the prosthetics and frequently raises the issue with the care team, aiming to convince them of its importance. The care professionals, however, perceive this as her making an issue out of something that, in their view, does not benefit Alma. They interpret Cara's insistence as a sign that she is struggling to emotionally process the changes associated with her mother's dementia. In doing so, they psychologize Cara's perspective, which enables them to dismiss it as irrelevant to Alma's current care needs.

As a response, the care professionals try to convince Cara that wearing the prosthetics is not in Alma's best interest. However, Cara cannot be convinced. As an advocate for her mother's well‐being, identity, and dignity, she continues to raise the issue, growing increasingly frustrated and emotional:

I came one day to change the laundry and things like that, and I saw that she didn't have her dentures in. I went looking: GONE!!! I searched everywhere: trash bins, laundry basket, etc. It's a real shame! […] I experience this as a lack of care and responsibility. Sorry. Now I have to call again about how and what to do with a new denture. But that will be the umpteenth one. And it's starting to become an “expensive joke”, that a person with dementia gets blamed for. I don't think this is fair, to be honest…

(journal entry, May 2021)

The care professionals, in turn, grow frustrated that Cara continues to raise this issue. They perceive her persistence as an active hindrance to Alma's well‐being, caused by Cara herself being unable to cope with the situation. This reflects their belief that the emotions of informal caregivers can impair their judgment. The care professionals begin to hold this against Cara:

“This is her way of expressing her dissatisfaction, because she is standing in her own way. It's probably not even about her mother; it's about her, it's her frustration, and she needs to find another way to deal with it. This has nothing to do with us”.

(Caroline, focus group, June 2021)

#### 3.2.2 Distancing Practices and a Tug of War Over Power

Cara and the care professionals all experience their collaborative relationship as strained. For the care professionals, the contact with Cara becomes overwhelming, as she continues to bring up topics they disagree on, and closely monitors the execution of care. Her dissatisfaction hangs them over the head:

Interviewer: “What would happen if there was more mutual understanding between you?”

Debbie: “It would give us some peace.”

(interview, March 2021)

In response to this, the care professionals shift their communication strategy from convincing to avoiding, to distance Cara and protect themselves. Issues that could lead to disagreement are avoided, for example, by omitting them from the daily care reports, which Cara can read as well:

Or, you know, when she's [Alma] sitting with us in the evening and she's eating some crisps, then some more, and then some more, we just won't write that down. Because otherwise, we'll get criticized [by Cara] for her having too much salt. So, things like that.

(Adriana, interview, March 2021)

Their avoidant strategy gives the care professionals more space to deliver care as they see fit. Although they tell Cara that they deliver care according to care agreements, they ultimately do what they believe is best for Alma. If they deviate from care agreements, they do not share this with Cara, preferring to avoid any issues with her.

The care professionals' avoidant strategy is aided by the COVID‐19 mitigation measures that were in place at the time, first prohibiting informal caregivers from entering the facility at all, and later, the facility's common rooms such as the kitchen and living area, where the care professionals often reside during their shift.

This avoidance limits opportunities for communication and insight into the care process for Cara. Resultingly, space for Cara's voice is further reduced, and the distance between Cara and Alma grows as well. Moreover, Cara perceives that COVID‐19 mitigation measures have been used as an excuse to avoid her. She feels like the care professionals are reluctant to engage with her.

At this point, a tug of war over power develops. Distanced from the care process, Cara frustratedly pushes to reduce this distance. Feeling shut out and powerless, she starts to cling to details in an attempt to regain some sense of control. She also admits that, on some level, she almost wishes something would go wrong. This would justify her concerns and show that they are based not on an inability to cope with her mother's illness, but on valid reasons:

I tell her [Alma] that she had her COVID vaccine today (“Hooray!”), a shot. She denies it. I repeat that she really did have it, along with all the residents in [the facility]! […] I immediately push aside a strange thought, that I would like to believe her when she says “I haven't been vaccinated.” I wasn't there, so I don't actually know. She is at the mercy of either reliable or unreliable care… Yeah, responsibility for life and death: pretty much handed over… Suppressed feeling of powerlessness.

(journal entry, January 2021)

However, Cara's attempts to reduce her distance to the care process further fuel the frustration of care professionals and their tendency to psychologize her perspective: “This daughter tackles every form of communication, as long as she can just unload her story” (Debbie, e‐mail, June 2021). Her persistence prompts the care professionals to fortify their position, maintaining distance as a way to protect themselves from emotional distress. In this way, the tug of war over power forms a downward spiral: the more Cara pushes to reduce the distance, the more frustrated the care professionals grow, reinforcing their need for distance.

#### 3.2.3 Pseudo‐Participation in the Multidisciplinary Care Team Meeting

In April 2021, a multidisciplinary care team meeting was held to discuss Alma's care. These meetings, typically held every 6 months, bring together professionals from various disciplines, alongside informal caregivers, to coordinate care and ensure a holistic approach.

For Cara, who desires to have more say in the care process, this meeting represents an ideal opportunity to give input. However, despite the intention of being a platform for collaboration, the meeting largely mirrors her previous interactions with care professionals. The usual one‐directional communication style is revisited as care professionals from various disciplines are united in convincing Cara of the benefit of pre‐made care decisions. Although each discipline is represented in the meeting by a single delegate, all are professionals, facing one informal caregiver, which creates a power‐imbalance. Moreover, the informal caregiver's input is relegated to the bottom of the meeting agenda, which results in too little time left to address all of Cara's concerns. Although she is included on paper, Cara finds herself silenced in practice, which fuels her frustration:

I felt a lot of resistance from them to agree with “my” opinions regarding my mother. They had the tendency to frame me as “disrespectful” with regard to my mother's autonomy. This made me feel bitter, and evoked a “fight‐response” within me; fighting to make clear that it wasn't about “my wish” but SPECIFICALLY my mother's wishes.

(journal entry, April 2021)

#### 3.2.4 Unwanted Feedback From Cara for the Care Professionals

Shortly after the multidisciplinary care team meeting about Alma, the care organization started an experiment with a digital feedback system for informal caregivers. With a desire to have more say in the care process, Cara sees an opportunity to do so through this system. It provides her with a channel for her input, which is still free from norms and protocols due to its experimental nature.

Cara takes this opportunity, leaving many messages in the system about concerns about care that she has had for a while, which she has tried to address with care professionals in other ways earlier. Her built‐up frustration results in her making sarcastic and cynical remarks in these messages.

Because of this feedback system, the possibility for care professionals to avoid Cara is diminished. The distance between them is abruptly reduced, and the sudden confrontation with Cara's feedback angers the care professionals. They resent Cara for sharing feedback about issues that have already been discussed or that happened a long time ago. Cara for instance shares feedback about the intake conversation, which she could not attend, showing that this issue has not been resolved for her after several years.

The care professionals particularly resent Cara for sharing feedback that, according to them, does not center on Alma's well‐being. This directly corresponds to their perspectives on care and collaboration, as described in part one of the Findings. As Alma's well‐being is central to their mission, the care professionals also feel attacked by Cara's feedback:

If it goes on like this, I'll be honest, with all of those complaints, I'm going to quit. They don't put you in a good mood, and just like Debbie said, it doesn't make your relationship with that family any better. […] I get really angry when I read all those messages from her.

(Caroline, focus group, June 2021)

The care professionals think that the only way to satisfy Cara is by aligning with her perspective on care. However, because they believe this perspective is not in Alma's best interest, agreeing with Cara would go against their core values. This puts them in a difficult position, which, coupled with their growing anger, compels them to further fortify, adopting an increasingly defensive stance towards Cara.

#### 3.2.5 Working Around the Conflict

The care professionals and Cara both reach a limit in their frustration. Their anger towards each other leads them to seek out others in the organizational system. They seemingly do this not with the intention to resolve the conflict, but rather to get the other party to stop exhibiting their “wrong” behavior.

For Cara, a limit is reached after the multidisciplinary care meeting. In an angry e‐mail to the facility's case manager, she outlines her feelings of outrage and exclusion:

I feel like I'm being hindered and not taken seriously in my role of helping my own mother. […] In my experience, it's impossible to collaborate with you in a normal way, as a relative, mentor, and first point of contact.

(e‐mail to the case manager, May 2021)

For the care professionals, a limit is reached after reading Cara's feedback in the experimental system. In response to this, they mobilize the location manager, who contacts Cara to correct her behavior on behalf of the team.

By seeking out others in the system, the care professionals and Cara work around the relational strains. Conversations about the care process and collaboration occur via intermediaries, but not between the care professionals and Cara themselves. These workarounds are not helpful in resolving the conflict. Rather, they leave it at an impasse.

#### 3.2.6 The End?

After the situations described here, we remained engaged with the facility for another year and a half. Although the intensity of the conflict decreased over time, the issue lingered, and the relationship between the care professionals and Cara remained strained. The dynamics highlighted here seem to mark their relationship, following a cycle of intensifying and calming down again, without resolving the underlying issues.

## 4 Discussion

This case study explored how relational tensions develop between a team of care professionals and an informal caregiver in a Dutch residential dementia care facility. The findings reveal a complex interplay of personal, relational, and organizational factors, embedded in broader structures of power.

### 4.1 Key Findings

At the personal level, findings indicate that professional and informal caregivers conceptualize good care differently. In this case study, we learned that professional caregivers adopt a mission‐driven, person‐centered approach to care. They tailor care to residents' current needs and wishes and emphasize their personal autonomy. This approach reflects dominant Western care policies, which promote person‐centered care as the moral standard (Dutch Care Institute 2024; O'Rourke et al. 2019), emphasizing identity and autonomy as core concepts (Lusk and Fater 2013). The case also shows that informal caregivers have a relationship‐centered approach to care. Their understanding of good care is not rooted in identity and autonomy, but rather in their loved one's life history and their emotional connection. Correspondingly, the ethical responsibilities of informal caregivers extend beyond supporting the resident as an individual; they also concern the preservation of their relationship. These divergent moral imperatives have been noted in previous research (Dohmen et al. 2025) and reflect a broader divide between healthcare and family ethics (Lindemann 2006). The resulting ethical mismatch not only hinders collaboration in practice but also points to a misalignment of current person‐centered care practices with the growing role of informal caregivers.

Relationally, the divergent conceptualizations of good care lead to friction in daily interactions. Rooted in different ethical systems, they cause repeated mutual violations of normative expectations. As described in moral conflict theory, such violations give rise to relational tensions (Michalski 2021). For example, while informal caregivers are typically motivated by their emotional bond with the resident, professional caregivers may expect a more emotionally detached approach. This expectation highlights a potential limitation of the person‐centered care framework: although it aims to prioritize the resident's well‐being, it can also distract from the resident's embeddedness in meaningful social relationships.

Also at the relational level, the case illustrates how tensions between professional and informal caregivers can escalate through a series of disruptive dynamics. To discuss these dynamics, we draw on Tronto's (1993) virtues of care: responsiveness, competence, responsibility, and attentiveness. These virtues are essential for fostering collaboration and mutual understanding in caregiving contexts (Tronto 1993). The first dynamic involves both parties advocating for their own views, seeking to convince rather than understand. This communication style signals a lack of responsiveness, as neither side truly engages with the other's needs, wishes, or concerns. The second dynamic concerns professional caregivers distancing informal caregivers from the care process, through avoidance and fortification, to protect their own caregiving approach and emotional well‐being. This reflects an absence of responsibility and competence, as professional caregivers neither seem to acknowledge relational challenges as part of their professional duties nor appear equipped to address them effectively. The resulting distance can fuel a third dynamic, in which informal caregivers, feeling excluded, attempt to reduce the distance. They may do so by closely monitoring care practices, appealing to a form of responsibility that professional caregivers do acknowledge: the duty to provide appropriate care for the resident. However, this move can spark a power struggle, with informal caregivers pushing for inclusion while professional caregivers resist. This tug‐of‐war then creates a downward spiral in the relationship, as it intensifies professional caregivers' frustrations, thereby reinforcing their need for distance. This reveals an absence of attentiveness. Neither side is able to recognize the other's needs, nor what is required for constructive collaboration. In the absence of these four virtues, both parties resort to workarounds, leaving the underlying conflict unresolved and ultimately undermining the quality of care (Tronto 1993).

At the organizational level, several factors seem to further entrench these disruptive dynamics. Processes of pseudo‐participation (inviting informal caregivers, but not meaningfully including them) create false expectations for informal caregivers, leading to relational strains (Michalski 2021). This indicates that simply organizing informal caregiver involvement is ineffective (or even counterproductive) if their contributions are not genuinely valued. Additionally, some organizational processes may enable the distancing of informal caregivers, such as infectious disease mitigation measures that limit contact with the outside world, or when superiors manage communication with informal caregivers on behalf of professional caregivers in case of relational tensions, thereby facilitating workarounds. When organizational processes allow for distancing, they also fail to support sustainable conflict resolution.

### 4.2 Power Structures

The dynamics between professional and informal caregivers are deeply shaped by power structures. Prior research has shown how the underestimation of residents' capacity to contribute (Novy et al. 2023), along with one‐sided organizational familiarity and support (Williams 2020), reinforces existing inequalities between professional caregivers and residents. Our findings suggest that these power asymmetries also extend to the relationship between professional and informal caregivers. Specifically, the results highlight how a powerful position enables professional caregivers to sideline informal caregivers' perspectives on good care. Positioned with less power, informal caregivers face a double bind: conform to remain included, or speak up and risk exclusion. This power asymmetry is echoed in previous studies (Dohmen et al. 2025; Hovenga et al. 2022) and resonates with moral conflict theory, which links social status imbalances to the emergence of conflict (Michalski 2021).

Importantly, these power‐related dynamics are not merely the result of individual actions, but are deeply rooted in systemic conditions (Tronto 2013; Williams 2020). Across many European countries, the long‐term care sector is under sustained pressure, placing professional caregivers in double binds as well. On the one hand, resource scarcity necessitates the involvement of informal caregivers. On the other hand, this involvement adds a new layer of complexity (i.e., managing collaboration) to their already heavy workload. Rather than reducing burdens, structural reforms such as these tend to increase the complexity of care and of the demands placed on professional caregivers (Baur et al. 2017). Operating within a context of chronic scarcity, they are confronted with situations where they cannot provide what they feel is necessary, leading to moral distress (Baur et al. 2017). This situation was likely intensified during the COVID‐19 pandemic, which introduced additional layers of scarcity and moral distress (Dohmen et al. 2022; Joo and Liu 2021). In an effort to navigate these situations, professional caregivers may resort to the path of least resistance (Williams 2020), for instance, avoiding collaboration with informal caregivers to minimize friction. This way of prioritizing manageability does not reflect a lack of commitment or care, but rather a depletion of resilience under increasing demands.

Tronto's (2013) fifth care virtue, solidarity, is particularly relevant here. Solidarity calls for a recognition of structural inequalities in caregiving and urges collective responsibility for addressing them. Without it, there is little room for mutual understanding or structural solutions; both parties are left managing tensions that stem not from personal failings, but from a system that does not sufficiently equip them for true collaboration.

### 4.3 Implications

Our findings imply that prevailing interpretations of person‐centered care fall short in supporting collaboration between professional and informal caregivers in residential dementia care. Scholars have previously raised concern that person‐centered care practices overemphasize autonomy, neglecting the interdependent nature of care relationships (Austin et al. 2009; Lloyd 2004; Nolan et al. 2004). Although person‐centered care frameworks do highlight the value of personal relationships and shared decision‐making (Hansson and Fröding 2021), and recent studies call for greater attention to relational ethics (O'Rourke et al. 2019) and reciprocity in care (Novy et al. 2023), these perspectives typically remain centered on the dyadic relationship between caregiver and care recipient. As a result, professional caregivers may feel able to fulfill their moral imperative (supporting the resident) without the participation of informal caregivers, or even experience this participation as a hindrance, rather than a resource.

To more effectively support collaboration between professional and informal caregivers, we argue that the moral imperative guiding professional caregivers must shift: from a focus on the individual resident and the traditional dyadic nature of care, towards a recognition of care as a relational practice involving a wider network (Nolan et al. 2004). Conceptualizing care as multidirectional, rather than bidirectional, acknowledges the agency and interdependence of all actors involved in the care process (Rockwell 2012). This shift not only supports a broader view of residents as embedded in networks of meaningful relationships, beneficial to their well‐being (Rockwell 2012), but also positions informal caregivers as integral partners to the care process, valuing their knowledge and emotional investment.

A reorientation towards relational care also has implications for the systemic power asymmetries that mark the relationship between professional caregivers, informal caregivers, and residents. Grounded in relational ethics, relational care holds that ethical practice emerges from the quality of the relationship between the actors involved, underscoring the need to address power issues to provide ethically sound care (Austin 2006). It foregrounds and embraces interdependency, relational personhood, dialogue, and community, and poses fundamental questions of power by recognizing experiential knowledge alongside professional knowledge, valuing professional and informal labor alike (Austin 2006; Austin et al. 2009). From a policy perspective, this proposed reorientation calls for the promotion of relational care as the moral standard guiding political and organizational choices, similar to the earlier paradigm shift from biomedical to person‐centered care. This way, efforts to enhance the participation of informal caregivers can fall on fertile ground.

For organizations, our findings offer insight into the dynamics that can deteriorate professional–informal caregiver relationships. Organizations should be alert to these dynamics and avoid unintentionally facilitating them. Instead, they should take proactive steps to facilitate the opposite, minimizing power asymmetries by enhancing organizational transparency, facilitating sustainable conflict resolution, and fostering mutual engagement, respect, trust, and recognition (Austin et al. 2009; Dohmen et al. 2025; Williams 2020). Practical implications following from our findings include redesigning information infrastructures to grant equal access to professional and informal caregivers, reducing professional‐driven decision‐making through collaborative agenda setting, and involving mediators to support conflict resolution.

Furthermore, organizations should take deliberate steps to embed ethical reflection into their processes and procedures. This can include initiatives such as organizing moral case deliberations or implementing tools like the ethical constellation tool, which helps visualize the moral landscape of the care process and sheds light on relationships, distance, and power (Baur et al. 2022; Van Dartel and Molewijk 2013). These ethical practices can be further supported through strengthening the moral and relational competencies of (future) professional caregivers in nursing education and training programs. This may include building skills in conflict management and meta‐communication, to enhance relational care practices (Abma and Baur 2015), and using personal experiences with care to foster dialogue and enhance reflection on the moral implications of professional practice (van den Eijnde et al. 2024). In light of this study, it may be particularly important to support (future) professional caregivers in ethically engaging with so‐called “less desirable” emotions, such as anger and antipathy, which are likely to arise from the tension and suffering inherent in caregiving, and may be further amplified in a system under chronic constraints (Baur et al. 2017).

These implications for nursing policy, practice, organization, and education may not only benefit collaboration between professional and informal caregivers but also support professional caregivers in providing quality care, ultimately supporting their well‐being, as well as that of residents and informal caregivers (Tronto 1993).

### 4.4 Study Limitations

A key limitation of this study is the absence of the resident perspective. This was shaped by practical constraints, such as COVID‐19 visiting restrictions that limited opportunities for face‐to‐face conversations with residents, and our reluctance to place additional demands on professional caregivers by asking them to facilitate remote interviews. Further, our initial focus for this case study was on the perspectives of professional and informal caregivers. However, as our understanding evolved, we came to see this focus as too narrow. Omitting the resident perspective leaves a critical gap in understanding collaborative relationships in residential dementia care.

The absence of the resident perspective also reflects a broader dynamic within the research context. Considering themselves the voice of the residents, the care professionals believed we could access the resident perspective through them, without having to burden the residents. Given that the broader research project relied heavily on the cooperation of professional caregivers, we were concerned that challenging this would jeopardize our relationship with the team. More fundamentally, this limitation reflects wider power asymmetries in care, society, and academic research (Groot et al. 2023). By not exploring the resident perspective, we risk silencing their voice and reinforcing the idea of residents as recipients of care, rather than active participants. This highlights the need for critical reflection on the structures that shape both care practices and knowledge production, and on the assumptions and compromises we make as researchers within those structures.

Other potential limitations of the study include its single‐case design, the focus on a highly strained relationship, and possible researcher bias. The single‐case design may limit the generalizability of findings, as the insights are based on a specific context with particular dynamics. To mitigate this, we aimed to enhance transferability by selecting a typical case and providing thick description, allowing readers to assess relevance for their own contexts (Abma and Stake 2014; Kroopnick 2013). The focus on a highly strained relationship may have led to findings that reflect more extreme or intensified relational dynamics than those in less conflicted situations, limiting the applicability of our findings. However, we also consider this a strength. The intensity of the case allowed us to explore relational dynamics in greater depth, which may still hold relevance for understanding disruptive dynamics across a broader range of situations. Nevertheless, the nature of the relationship should be kept in mind when interpreting the findings. Lastly, possible researcher bias deserves attention. While our prolonged engagement in the study context brought valuable insights, it may also have shaped the research through the relationships we developed in practice. Despite our efforts to remain reflexive and limit unwanted influence, it is possible that our assumptions and positionalities affected both data collection and analysis.

### 4.5 Future Research

A key limitation of this study is the absence of the resident perspective. Future research should address this by adopting methods that enable more meaningful inclusion of residents, such as taking more time than usual to build rapport and using observational or visual methods (Webb et al. 2020). Observational studies hold particular promise, especially when extended to include triadic interactions between residents, professional caregivers, and informal caregivers. As Tolhurst et al. (2017) noted, caregiving is too often examined as a set of parallel individual experiences, rather than as an interactional process. Observational methods not only help to foreground the resident's experience, but also make it possible to study care as a multidirectional, co‐constructed process, revealing relational dynamics that may remain hidden in more fragmented approaches.

In addition, the focus of this study on a highly strained relationship invites complementary research into more positive or effective collaborative caregiving relationships. Exploring which personal, relational, and organizational factors enable these relationships to flourish can provide valuable insight into further supporting collaboration in the complex context of residential dementia care, both individually and collectively.

Finally, future research should explore how education on moral and relational skills can support collaborative nursing care practices. This includes both the development of such programs and the evaluation of their effectiveness. Investigating how education and training can help shape caregivers' moral reasoning, sensitivity to relational dynamics, and practical actions could be crucial in supporting sustainable, ethical care relationships in complex and evolving care contexts.

Taken together, these directions for future research can contribute to a more nuanced and hopeful understanding of collaboration in residential dementia care. By including resident voices, learning from effective practices, and investing in ethical and relational competencies, we can not only deepen our understanding of collaborative challenges but also open up new possibilities for more equitable and relationally responsive care.

## Ethics Statement

The study was reviewed and approved by the Institutional Review Board of the Medical Ethical Committee Leiden‐Den Haag‐Delft for observational studies. The study was conducted in accordance with the Declaration of Helsinki.

## Consent

Informed consent for participation and publication was obtained from all participants involved in the study.

## Conflicts of Interest

The authors declare no conflicts of interest.

## Data Availability

The data that support the findings of this study are available on request from the corresponding author. The data are not publicly available due to privacy or ethical restrictions.
